# Lung adenocarcinoma diagnosed incidentally after renal biopsy for suspected right renal cancer

**DOI:** 10.1093/jscr/rjab092

**Published:** 2021-04-06

**Authors:** Sat Prasad Nepal, Takeshi Shichijo, Yoshio Ogawa, Takehiko Nakasato, Yoshihiro Nakagami, Jun Morita, Kazuhiko Oshinomi, Yoshiko Maeda, Tsutomu Unoki, Tatsuki Inoue, Ryosuke Kato, Satoshi Amano, Moyuru Mizunuma

**Affiliations:** Showa University School of Medicine, Department of Urology, Tokyo 142-8555, Japan

## Abstract

We present a case of lung adenocarcinoma metastasizing to the right clear cell renal cell carcinoma diagnosed by computed tomography (CT)-guided renal biopsy and immunohistochemistry. A 72-year-old male patient had right lower abdominal pain for 3 days, followed by right loin pain for 10 days. On CT scan, renal cell cancer was suspected with multiple metastases. Renal cell cancer with metastatic lung adenocarcinoma was diagnosed on CT-guided renal biopsy with positive immunohistochemical markers. The patient, unfortunately, expired after few days of diagnosis. Tumor-to-tumor metastasis is an unusual disease, and its tumors are aggressive. A definite diagnosis of tumor-to-tumor metastasis is a clinical challenge. Immunohistochemistry helped us in the diagnosis without the primary lesion biopsy.

## INTRODUCTION

Tumor-to-tumor metastasis is an incredibly unique phenomenon, identified using the following criteria: (1) presence of two or more distinct tumors; (2) the tumor should not be a lymph node involved in lymphoreticular tumors; (3) extravascular metastasis and (4) not a collision tumor (two distinct tumors connected by a transitional zone) [1, 2]. In these cases, renal cancer is the most common recipient, whereas lung cancer is the most common donor [2, 3]. Majority of cases are diagnosed in autopsy [2].

## CASE REPORT

A 72-year-old patient presented with continuous pain in the right lower abdomen and right lower ribs (around the 11th rib) for 3 days, followed by right loin and back pain for 10 days. The patient took painkillers with sleeping pills for pain relief, but this was ineffective, prompting consult to our hospital.

He has a history of hypertension and gout, managed with amlodipine, febxostat, ethyl eicosapentaenoic acid, magnesium oxide and brotizolam. He also has 50 pack-year smoking history. The patient has undergone an appendectomy and also had a right putamen hemorrhage 4 years ago. His father had lung cancer, whereas his mother had laryngeal cancer.

On arrival, his C-reactive protein was elevated (2.84), and urine cytology was Class II. Other blood examinations were within normal limits.

**Figure 1:**
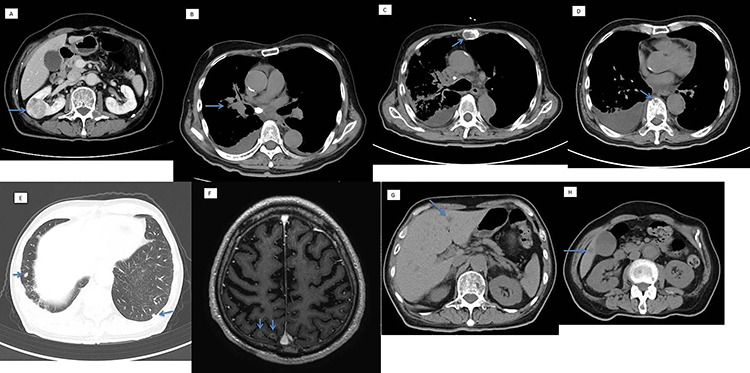
Computed tomography scan showing (**A**) a 38-mm right renal mass (suspected renal cancer), (**B**) right hilar lung cancer, with metastasis in the (**C** and **D**) bone, (**E**) lungs, (**F**) brain and (**G**) liver.

On computed tomography (CT) scan, there was a 38-mm right renal mass, giving an impression of renal cancer, right hilar lung tumor, and bone, lungs, brain, and, liver metastases, with right pleural effusion (Fig. 1). We wanted to check if the renal mass was a primary renal cell cancer or metastatic; a CT-guided renal biopsy was then planned.

**Figure 2:**
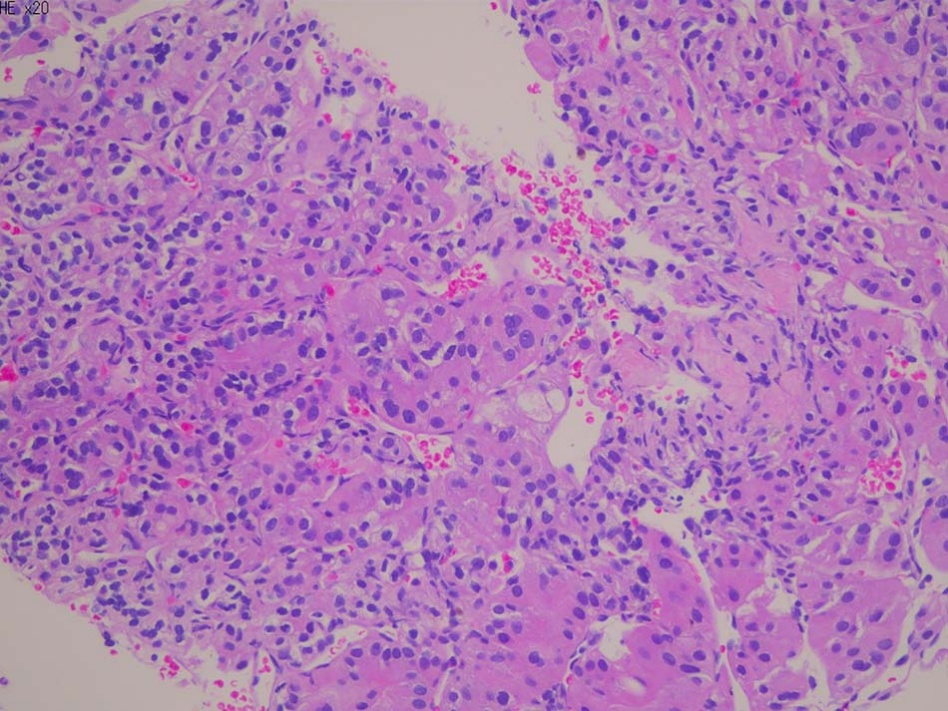
Histology showed a proliferation of large nucleated atypical cells and eosinophilic cytoplasm. Some multinucleated cells with distinct nucleolus are scattered.

We found a clear cell renal cell cancer with a metastatic lung adenocarcinoma on CT-guided renal biopsy (Fig. 2). We were able to diagnose lung cancer from renal biopsy by immunohistochemical staining (Thyroid transcription factor 1 [TTF-1] and Napsin positive) without the primary lung tumor biopsy. The tumor was also positive for the following immunohistochemical markers: vimentin, cluster of differentiation 10 (CD10), heat shock protein 70, Glypcian-3, hepatocyte-specific antigen and paired-box gene 8.

A bone scan index, with a value of 0.62, revealed nine hot spot lesions on the thoracic and lumbar vertebra and ilium.

After 3 days of admission, the patient’s oxygen saturation started dropping. White blood cell count, lactate dehydrogenase, alkaline phosphatase, aspartate transaminase and D-dimers were all elevated. The patient unfortunately expired 10 days later due to pneumonia and sepsis.

## DISCUSSION

What makes our case unique is that lung cancer was first discovered incidentally through chest CT scan and immunohistology of renal biopsy, before the onset of any classic symptoms of lung adenocarcinoma (continuous cough, bloody sputum, shortness of breath and weight loss). Moreover, clinical diagnosis of lung tumor to kidney tumor metastasis is extremely uncommon [1, 2, 4–8] (Table 1).

**Table 1:** Relevant literature on lung cancer metastatic to renal cell carcinoma

SN	Author (Year)	Age/Sex	Lung cancer histology	RCC location
1	Schmorl (1928)^a^			
2	Walther (1948)^a^	47 M		Lt
3	Rabson (1954)^a^	58 M		Lt
4	Rabson (1954)^a^	65 M	Mucoid adenocarcinoma	Lt
5 6	Boyd (1955) (2 cases)^a^			
7	Dobbing (1958)^a^	71 M		Lt
8	Wheelock (1962)^a^			
9	Moerterl (1966)^a^			
10	Campbell (1968)^a^	74 M	Bronchogenic ca	Rt
11	Ottoson L (1968)^b^	74	Adenocarcinoma	
12	Ottoson L (1968)^b^	77	Oat cell bronchial ca	
13	Maloney (1968)^b^	59 M	Undifferentiated lung ca	Rt
14	Sharma (1969)^b^	59 M	Undifferentiated small cell ca	Rt
15	Ichijima (1980)^b^	41 F	Moderately differentiated Lung adenocarcinoma	Lt
16	Shuangshoti (1983)^b^	79 F	Poorly differentiated adenocarcinoma	Lt
17	Sella (1987)^b^	56 F	Poorly differentiated adenocarcinoma	Rt
18	Hibi (1991)	62 M	Oat cell carcinoma of lung	Lt
19	Granville (2005)^b^	65 F	Moderately differentiated adenocarcinoma	Lt
20	Sawada (2009)^b^	97 F	Poorly differentiated Lung adenocarcinoma	Lt
21	Duprez (2009)	60 M	Neuroendocrine lung cancer	Lt
22	Aggarwal N (2012)	57 M	Non-small cell Lung ca	Lt
23	Matsukuma S (2013)	88 M	Lung adenocarcinoma	Lt
24	Matsukuma S (2013)	69 M	Lung adenocarcinoma	Rt
25	Matsukuma S (2013)	72 M	Lung adenocarcinoma	Rt
26	Matsukuma S (2013)	48 M	Lung adenocarcinoma	Lt
27	Matsukuma S (2013)	82 M	Small cell lung carcinoma	Lt
28	Huang H (2016)		Lung adenocarcinoma	
29	Our case (2021)	72 M	Lung adenocarcinoma	Rt

^a^Review of Campbell *et al*. [1].

^b^Review of Sawada *et al*. [4].

Empty spaces means unknown; M: male, F: female; RCC: renal cell carcinoma; Lt: left, Rt: right.

In tumor-to-tumor metastasis, lung cancer is the most common donor, whereas kidney cancer is the most common recipient [3]. The pathogenesis for this is still being studied. The ‘seed and soil’ theory speculates that this is due to the recipient’s hypervascularity, making it easier for circulating tumor cells to arrive from the donor and because the recipient cells form a suitable environment for donor cells [2]. Kidney’s vascularity and glycogen and lipid richness cause it to be a favorable recipient of metastatic tumor [1, 9].

Immunohistology aided us in identifying markers of lung adenocarcinoma metastasis (TTF-1 and Napsin) without the primary lung tumor biopsy.

Tumor-to-tumor metastasis is aggressive in nature and carries a poor prognosis [2]. Our patient presented with multiple lung, brain and liver metastases, suggesting its aggressive nature.

We could not start anti-cancer treatment for the patient as he had reduced oxygen saturation, after which he regrettably expired.

In conclusion, we present a rare clinical case of lung to kidney, tumor-to-tumor metastasis diagnosed by CT-guided renal biopsy and immunohistochemistry. This condition is hard to diagnose clinically and has a poor prognosis.

## CONSENT FOR THE ARTICLE

Written informed consent was obtained from the patient’s next of kin for publication of this case report and any accompanying images.

## CONFLICT OF INTEREST STATEMENT

None declared.

## FUNDING

None.
